# Supplementary material for: Characteristic of persistent human papillomavirus infection in women worldwide: a meta–analysis
Source: PeerJ. 2023 Nov 14;11:e16247. doi: 10.7717/peerj.16247 (PMC10655709; doi:10.7717/peerj.16247)
Supplement: Supplemental Information 2 [file peerj-11-16247-s002.docx]

Meta Analysis

data <- transform(rate,p=rate$Case/rate$Number)

shapiro.test(data$p)

names(data)

hist(data$p)rate1<-transform(rate,p=rate$Case/rate$Number,

log=log(rate$Case/rate$Number),

logit=log((rate$Case/rate$Number)/(1-rate$Case/rate$Number)),

arcsin.size=asin(sqrt(rate$Case/(rate$Number+1))),

darcsin=0.5*(asin(sqrt(rate$Case/(rate$Number+1)))+asin((sqrt(rate$Case+1)/(rate$Number+1)))))

head(rate1)

shapiro.test(rate1$p)

shapiro.test(rate1$log)

shapiro.test(rate1$logit)

shapiro.test(rate1$arcsin)

shapiro.test(rate1$darcsin)

hist(rate1$logit)

hist(rate1$log)

hist(rate1$arcsin)

hist(rate1$darcsin)library(meta)meta<-metaprop(Case,Number,data=rate,

studlab=paste(rate$Author,rate$Year,sep="-"),sm="PLOGIT")

summary(meta)forest(meta,col.square="blue",col.diamond="red")funnel(meta,comb.random=TRUE)

metabias(meta,method.bias="linreg",plotit=T,k.min=10)metainf(meta,pooled='random')forest(metainf(meta,pooled="random"))meta1<metaprop(Case,Number,data=rate,studlab=paste(rate$Author,rate$Year,sep="-"),sm="PLOGIT",subgroup =Area)

summary(meta1)forest(meta1,col.square="blue",col.diamond="red",plotwidth="10cm",colgap.forest.left="1cm",colgap.forest.right="1cm",just.forest="right",colgap.left="1cm",colgap.right = "1cm")metareg(meta1,sample+time+score+hr+year+area,method="ML")
