# Supplementary material for: Characteristic of persistent human papillomavirus infection in women worldwide: a meta–analysis
Source: PeerJ. 2023 Nov 14;11:e16247. doi: 10.7717/peerj.16247 (PMC10655709; doi:10.7717/peerj.16247)
Supplement: Supplemental Information 3 [file peerj-11-16247-s003.docx]

Table S1. Agency for Healthcare Research and Quality for assessing the quality of studies in the meta-analysis

| Author | Year | A | B | C | D | E | F | G | H | I | J | K | Quality score |
| --- | --- | --- | --- | --- | --- | --- | --- | --- | --- | --- | --- | --- | --- |
| Xu Y.L. | 2021 | 1 | 1 | 1 | 1 | 0 | 1 | 0 | 0 | 0 | 0 | 1 | 6 |
| Jin R. | 2020 | 1 | 1 | 1 | 1 | 0 | 1 | 0 | 1 | 0 | 1 | 1 | 8 |
| Wei D.L. | 2019 | 1 | 1 | 1 | 1 | 0 | 1 | 0 | 0 | 0 | 0 | 1 | 6 |
| Zhong X.M. | 2018 | 1 | 1 | 1 | 1 | 0 | 1 | 0 | 0 | 0 | 1 | 1 | 7 |
| Xu S.S. | 2018 | 1 | 1 | 1 | 1 | 0 | 1 | 1 | 1 | 0 | 1 | 1 | 9 |
| Shen L.J. | 2018 | 1 | 1 | 1 | 1 | 0 | 1 | 0 | 1 | 0 | 1 | 1 | 8 |
| Wang X.Q. | 2014 | 1 | 1 | 1 | 1 | 0 | 1 | 0 | 0 | 0 | 1 | 1 | 7 |
| Hu M.M. | 2021 | 1 | 0 | 1 | 1 | 0 | 1 | 0 | 0 | 0 | 1 | 1 | 6 |
| Zhang Q. | 2015 | 1 | 0 | 1 | 1 | 0 | 1 | 0 | 0 | 0 | 1 | 1 | 6 |
| Long X. | 2020 | 1 | 1 | 1 | 1 | 0 | 1 | 0 | 0 | 0 | 1 | 1 | 7 |
| Li M. | 2021 | 1 | 1 | 1 | 1 | 0 | 1 | 0 | 0 | 0 | 1 | 1 | 7 |
| Ingabire C. | 2018 | 1 | 1 | 1 | 1 | 0 | 1 | 0 | 1 | 0 | 1 | 1 | 8 |
| Liu J. | 2020 | 1 | 1 | 1 | 1 | 0 | 1 | 0 | 1 | 0 | 1 | 1 | 8 |
| Nielsen A. | 2010 | 1 | 1 | 1 | 1 | 0 | 1 | 0 | 1 | 0 | 0 | 1 | 7 |
| Sammarco M. L. | 2013 | 1 | 0 | 1 | 1 | 0 | 1 | 0 | 0 | 0 | 1 | 1 | 6 |
| Stensen S. | 2016 | 1 | 1 | 1 | 1 | 0 | 1 | 1 | 1 | 0 | 0 | 1 | 8 |
| Li N. | 2017 | 1 | 1 | 1 | 1 | 0 | 1 | 0 | 1 | 0 | 1 | 1 | 8 |
| Miranda P. M. | 2013 | 1 | 1 | 1 | 0 | 0 | 1 | 0 | 0 | 0 | 1 | 1 | 6 |
| Cuschieri K. S. | 2005 | 1 | 0 | 1 | 0 | 0 | 1 | 0 | 1 | 0 | 0 | 1 | 5 |
| Sycuro L. K. | 2008 | 1 | 0 | 1 | 1 | 0 | 1 | 0 | 1 | 0 | 0 | 1 | 6 |
| Schmeink C. E. | 2011 | 1 | 1 | 1 | 1 | 0 | 1 | 0 | 1 | 0 | 1 | 1 | 8 |
| Muwonge R. | 2020 | 1 | 0 | 1 | 1 | 0 | 1 | 0 | 0 | 0 | 0 | 1 | 5 |
| Ye J. | 2010 | 1 | 1 | 1 | 1 | 0 | 1 | 0 | 1 | 0 | 1 | 1 | 8 |
| Soto-De León S. C. | 2014 | 1 | 1 | 1 | 1 | 0 | 1 | 0 | 1 | 0 | 1 | 1 | 8 |
| Lai C. H. | 2008 | 1 | 1 | 1 | 1 | 0 | 1 | 0 | 0 | 0 | 1 | 1 | 7 |
| Richardson H. | 2003 | 1 | 1 | 1 | 1 | 0 | 1 | 0 | 0 | 0 | 1 | 1 | 7 |
| Bulkmans N. W. J. | 2007 | 1 | 1 | 1 | 1 | 0 | 1 | 0 | 0 | 0 | 1 | 1 | 7 |
| Krings A. | 2019 | 1 | 1 | 1 | 1 | 0 | 1 | 0 | 0 | 0 | 1 | 1 | 7 |

A. Define the source of information (survey, record review)

B. List inclusion and exclusion criteria for exposed and unexposed subjects (cases and controls) or refer to previous publications

C. Indicate time period used for identifying patients

D. Indicate whether or not subjects were consecutive if not population-based

E. Indicate if evaluators of subjective components of study were masked to other aspects of the status of the participants

F. Describe any assessments undertaken for quality assurance purposes (e.g., test/retest of primary outcome measurements)

G. Explain any patient exclusions from analysis

H. Describe how confounding was assessed and/or controlled.

I. If applicable, explain how missing data were handled in the analysis

J. Summarize patient response rates and completeness of data collection

K. Clarify what follow-up, if any, was expected and the percentage of patients for which incomplete data or follow-up was obtained
