# Supplementary material for: Characteristic of persistent human papillomavirus infection in women worldwide: a meta–analysis
Source: PeerJ. 2023 Nov 14;11:e16247. doi: 10.7717/peerj.16247 (PMC10655709; doi:10.7717/peerj.16247)
Supplement: Supplemental Information 4 [file peerj-11-16247-s004.docx]

**1.PubMed**

("Persistent Infection"[MeSH Terms] OR "infection persistent"[Title/Abstract] OR "persistent infections"[Title/Abstract] OR "long term infection"[Title/Abstract] OR "infection long term"[Title/Abstract] OR "long term infections"[Title/Abstract] OR "chronic infection"[Title/Abstract] OR "chronic infections"[Title/Abstract] OR "infection chronic"[Title/Abstract] OR "persistence"[Title/Abstract]) AND ("Alphapapillomavirus"[MeSH Terms] OR "hpv human papillomavirus"[Title/Abstract] OR "hpv human papillomaviruses"[Title/Abstract] OR "human papillomavirus hpv"[Title/Abstract] OR "human papillomaviruses hpv"[Title/Abstract] OR "human papillomavirus"[Title/Abstract] OR "human papillomaviruses"[Title/Abstract] OR "papillomavirus human"[Title/Abstract] OR "papillomaviruses human"[Title/Abstract] OR "HPV"[Title/Abstract])

**2.Embase**

#1:'wart virus'/exp

#2:'chronic infection'/exp

#3:'hpv human papillomavirus':ab,ti OR 'hpv human papillomaviruses':ab,ti OR 'human papillomavirus, hpv':ab,ti OR 'human papillomaviruses, hpv':ab,ti OR 'human papillomavirus':ab,ti OR 'human papillomaviruses':ab,ti OR 'papillomavirus, human':ab,ti OR 'papillomaviruses, human':ab,ti OR hpv:ab,ti

#4:persistence:ab,ti OR 'infection, persistent':ab,ti OR 'persistent infections':ab,ti OR 'long term infection':ab,ti OR 'infection, long term':ab,ti OR 'long term infections':ab,ti OR 'chronic infection':ab,ti OR 'chronic infections':ab,ti OR 'infection, chronic':ab,ti

#1 OR #3

#2 OR #4

#5 AND #6

**3.Cochrane**

#1:("Human Papillomavirus"):ti,ab,kw OR ("HPV Human Papillomavirus"):ti,ab,kw OR ("HPV Human Papillomaviruses"):ti,ab,kw OR ("Papillomavirus, Human"):ti,ab,kw OR ("Human Papillomavirus, HPV"):ti,ab,kw OR ("Human Papillomaviruses, HPV"):ti,ab,kw OR ("Papillomaviruses, Human"):ti,ab,kw OR ("Human Papillomaviruses"):ti,ab,kw OR (Alphapapillomaviruses):ti,ab,kw OR (HPV):ti,ab,k

#2:("Long Term Infection"):ti,ab,kw OR ("Persistent Infections"):ti,ab,kw OR ("Infection, Long Term"):ti,ab,kw OR ("Infection, Persistent"):ti,ab,kw OR ("Long Term Infections"):ti,ab,kw OR ("Infection, Chronic"):ti,ab,kw OR ("Chronic Infections"):ti,ab,kw OR ("Chronic Infection"):ti,ab,kw OR ("Persistence"):ti,ab,kw

#1And#2

**4.CNKI**

#1: HPV + human papillomavirus

#2: Persistent infection

#1 And #2

**5.Wan Fang**

#1: HPV or human papillomavirus or Cervical cancer

#2: Persistent infection or HPV infection

#1 And #2

**6.VIP**

#1: HPV or human papillomavirus or Cervical cancer

#2: Persistent infection or HPV infection

#1 And #2
