# Supplementary material for: Characteristic of persistent human papillomavirus infection in women worldwide: a meta–analysis
Source: PeerJ. 2023 Nov 14;11:e16247. doi: 10.7717/peerj.16247 (PMC10655709; doi:10.7717/peerj.16247)
Supplement: Supplemental Information 6 [file peerj-11-16247-s006.docx]

Systematic review

Please select one of the options below to edit your record. Either option will create a new version of the record - the existing version will remain unchanged.

A list of fields that can be edited in an update can be found here

1. * Review title.

Give the title of the review in English

Characterize of Persistent Human Papillomavirus Infection in Women: A Meta -Analysis

1. Original language title.

For reviews in languages other than English, give the title in the original language. This will be displayed with the English language title.

1. * Anticipated or actual start date.

Give the date the systematic review started or is expected to start.

18/02/2022

1. * Anticipated completion date.

Give the date by which the review is expected to be completed.

15/07/2022

1. * Stage of review at time of this submission.

**This field uses answers to initial screening questions. It cannot be edited until after registration.**

Tick the boxes to show which review tasks have been started and which have been completed.

Update this field each time any amendments are made to a published record.

The review has not yet started: No

| **Review stage** | **Started** | **Completed** |
| --- | --- | --- |
| Preliminary searches | Yes | No |
| Piloting of the study selection process | No | No |
| Formal screening of search results against eligibility criteria | No | No |
| Data extraction | No | No |
| Risk of bias (quality) assessment | No | No |
| Data analysis | No | No |

Provide any other relevant information about the stage of the review here.

1. * Named contact.

The named contact is the guarantor for the accuracy of the information in the register record. This may be any member of the review team.

Ming Zhao

Email salutation (e.g. "Dr Smith" or "Joanne") for correspondence:

Miss Zhao

1. * Named contact email.

Give the electronic email address of the named contact

[zhaoming129166@163.com](mailto:zhaoming129166@163.com)

1. Named contact address

**PLEASE NOTE this information will be published in the PROSPERO record so please do not enter private**

**information, i.e. personal home address**

Give the full institutional/organisational postal address for the named contact.

Yongan Avenue, Yunhe District, Cangzhou City, Hebei Province

1. Named contact phone number.

Give the telephone number for the named contact, including international dialling code.

13127316690

1. * Organisational affiliation of the review.

Full title of the organisational affiliations for this review and website address if available. This field may be completed as 'None' if the review is not affiliated to any organisation.

Jiamusi University

Organisation web address:

1. * Review team members and their organisational affiliations.

Give the personal details and the organisational affiliations of each member of the review team. Affiliation refers to groups or organisations to which review team members belong.

**NOTE: email and country now MUST be entered for each person, unless you are amending a published record.**

Miss Ming Zhao. Jiamusi University

Limei Luo. Shandong Maternal and Child Health Care Hospital

1. * Funding sources/sponsors.

Details of the individuals, organizations, groups, companies or other legal entities who have funded or sponsored the review.

None

State the funder; grant or award number and the date of award

1. * Conflicts of interest.

List actual or perceived conflicts of interest (financial or academic).

None

1. Collaborators.

Give the name and affiliation of any individuals or organisations who are working on the review but who are not listed as review team members. **NOTE: email and country must be completed for each person, unless you are amending a published record.**

1. * Review question.

State the review question(s) clearly and precisely. It may be appropriate to break very broad questions down into a series of related more specific questions. Questions may be framed or refined using PI(E)COS or similar where relevant

1. The incidence of persistent infection of various subtypes of HPV 2.HPV persistent infection rate 3.Multiple, single persistent infection rates of HPV 4.Persistent HPV infection rates in different age groups
2. * Searches.

State the sources that will be searched (e.g. Medline). Give the search dates, and any restrictions (e.g. language or publication date). Do NOT enter the full search strategy (it may be provided as a link or attachment below.)

We will search the following databases:

1. PubMed
2. Embase
3. Cochrane
4. CNKI (China National Knowledge Infrastructure)
5. Wanfang database
6. VIP Database of Chinese Sci-tech Journals

Search period: From the date of construction to February 28, 2022.

Language:Chinese or English

1. URL to search strategy.

Upload a file with your search strategy, or an example of a search strategy for a specific database, (including the keywords) in pdf or word format. In doing so you are consenting to the file being made publicly accessible.

Or provide a URL or link to the strategy. Do NOT provide links to your search **results.**

Do not make this file publicly available until the review is complete

1. * Condition or domain being studied.

Give a short description of the disease, condition or healthcare domain being studied in your systematic review.

The main healthcare domain being evaluated is cervical cancer prevention.Human papillomavirus is a necessary cause of cervical cancer.We further optimize cervical cancer prevention strategies by analyzing the distribution and prevalence of persistent HPV infection in women around the world, and provide reference for cervical cancer prevention and HPV vaccine development.

1. * Participants/population.

Specify the participants or populations being studied in the review. The preferred format includes details of both inclusion and exclusion criteria.

HPV positive infection.Pregnancy or lactation, autoimmune diseases, cervical intraepithelial neoplasia and conical resection, cervical lesions, and other microbial infection were excluded

1. * lntervention(s)_f_ exposure(s)<

Give full and clear descriptions or definitions of the interventions or the exposures to be reviewed. The preferred format includes details of both inclusion and exclusion criteria.

This is not a review of interventions.

1. * Comparator(s)/control.

Where relevant, give details of the alternatives against which the intervention/exposure will be compared (e.g. another intervention or a non-exposed control group). The preferred format includes details of both inclusion and exclusion criteria.

None

1. * Types of study to be included.

Give details of the study designs (e.g. RCT) that are eligible for inclusion in the review. The preferred format includes both inclusion and exclusion criteria. If there are no restrictions on the types of study, this should be stated.

Inclusion criteria: cross-sectional study.

Exclusion criteria: review, conference reports and other literature

1. Context.

Give summary details of the setting or other relevant characteristics, which help define the inclusion or exclusion criteria.

1. * Main outcome(s).

Give the pre-specified main (most important) outcomes of the review, including details of how the outcome is defined and measured and when these measurement are made, if these are part of the review inclusion criteria.

The incidence of persistent infection of various subtypes of HPV

Measures of effect

Rate( %)

1. * Additional outcome⑸.

List the pre-specified additional outcomes of the review, with *a* similar level of detail to that required for main outcomes. Where there are no additional outcomes please state 'None' or 'Not applicable' as appropriate to the review

HPV persistent infection rate、Persistent HPV infection rates in different age groups. Multiple, single persistent infection rates of HPV

Measures of effect

Rate( %)

1. * Data extraction (selection and coding).

Describe how studies will be selected for inclusion. State what data will be extracted or obtained. State how this will be done and recorded.

One researcher will review the literature and two researchers will independently extract the data.Should any disagreements arise, they will be discussed and if need be, they will be resolved by a third party.The following information is mainly extracted from each literature: (1) Basic information of the included studies, including author; publication time and region; (2) Baseline characteristics of the subjects: number of HPV positive cases, number of HPV positive subtypes, number of HPV positive single multiple infections, and number of HPV positive in different age groups;(3) Outcome indicators: HPV persistent infection rate, HPV subtype persistent infection rate, HPV persistent infection rate at all ages, single multiple infection persistent infection rate

1. * Risk of bias (quality) assessment.

State which characteristics of the studies will be assessed and/or any formal risk of bias/quality assessment tools that will be used.

A critical appraisal of included studies will be carried out independently by two individuals.ln this study, the quality of the literature was assessed according to the quality assessment criteria recommended by the Agency for Healthcare Research and Quality (AHRQ) for cross-sectional studies.

1. * Strategy for data synthesis.

Describe the methods you plan to use to synthesise data. This **must not be generic text** but should be **specific to your review** and describe how the proposed approach will be applied to your data.

If meta-analysis is planned, describe the models to be used, methods to explore statistical heterogeneity, and software package to be used.

The results of each study will be analyzed through a meta-analysis, using random-effects models to plot forest plots. The source of heterogeneity was determined by subgroup analysis, sensitivity analysis and meta-regression analysis. Publication bias was evaluated by funnel plot and Egger linear regression analysis. Statistical analysis was performed using R software.

1. * Analysis of subgroups or subsets.

State any planned investigation of 'subgroups' . Be clear and specific about which type of study or participant will be included in each group or covariate investigated. State the planned analytic approach.

Subgroup analysis will be performed for different groups, including: region, sample size, follow-up time, sample source, quality score, and year of publication

1. * Type and method of review.

Select the type of review, review method and health area from the lists below.

Type of review

Cost effectiveness No

Diagnostic No

Epidemiologic No

Individual patient data (IPD) meta-analysis No

Intervention No

| Living systematic review | No |
| --- | --- |
| Meta-analysis | No |
| Methodology | No |
| Narrative synthesis | No |
| Network meta-analysis | No |
| Pre-clinical | No |
| Prevention | No |
| Prognostic | No |

Prospective meta-analysis (PMA)

Review of reviews No

Service delivery No

Synthesis of qualitative studies No

Systematic review Yes

Other No

Health area of the review

Alcohol/substance misuse/abuse No

No

Blood and immune system No

Cancer No

Cardiovascular No

Care of the elderly No

Child health No

Complementary therapies No

COVID-19 No

Crime and justice No

Dental No

Digestive system No

Ear; nose and throat No

Education No

Endocrine and metabolic disorders No

Eye disorders No

General interest No

Genetics No

Health inequalities/health equity No

Infections and infestations No

International development No

Mental health and behavioural conditions No

Musculoskeletal No

Neurological No

Nursing No

Obstetrics and gynaecology Yes

Oral health No

Palliative care No

Perioperative care No

Physiotherapy No

Pregnancy and childbirth No

Public health (including social determinants of

health) Yes

Rehabilitation No

Respiratory disorders No

Service delivery No

Skin disorders No

Social care No

Surgery No

Tropical Medicine No

Urological No

Wounds, injuries and accidents No

Violence and abuse No

1. Language.

Select each language individually to add it to the list below, use the bin icon to remove any added in error.

English

There is not an English language summary

1. * Country.

Select the country in which the review is being carried out For multi-national collaborations select all the countries involved.

China

1. Other registration details.

Name any other organisation where the systematic review title or protocol is registered (e.g. Campbell, or The Joanna Briggs Institute) together with any unique identification number assigned by them.

If extracted data will be stored and made available through a repository such as the Systematic Review Data Repository (SRDR), details and a link should be included here. If none, leave blank.

1. Reference and/or URL for published protocol.

If the protocol for this review is published provide details (authors, title and journal details, preferably in Vancouver format)

No I do not make this file publicly available until the review is complete

1. Dissemination plans.

Do you intend to publish the review on completion?

No

1. Keywords.

Give words or phrases that best describe the review. Separate keywords with a semicolon or new line. Keywords help PROSPERO users find your review (keywords do not appear in the public record but are included in searches). Be as specific and precise as possible. Avoid acronyms and abbreviations unless these are in wide use.

1. Details of any existing review of the same topic by the same authors.

If you are registering an update of an existing review give details of the earlier versions and include a full bibliographic reference, if available.

1. * Current review status.

Update review status when the review is completed and when it is published.

New registrations must be ongoing so this field is not editable for initial submission.

ReviewOngoing

1. Any additional information.

Provide any other information relevant to the registration of this review.

1. Details of final report/publication(s) or preprints if available.

Leave empty until publication details are available OR you have a link to a preprint (NOTE: this field is not editable for initial submission).

List authors, title and journal details preferably in Vancouver format.
