# Supplementary material for: Characteristic of persistent human papillomavirus infection in women worldwide: a meta–analysis
Source: PeerJ. 2023 Nov 14;11:e16247. doi: 10.7717/peerj.16247 (PMC10655709; doi:10.7717/peerj.16247)
Supplement: Supplemental Information 7 [file peerj-11-16247-s007.docx]

Cervical cancer (CC) is a common malignant tumor, and ranks the fourth in the incidence of female malignant tumors in the world. It is the only cancer with definite etiology that can be controlled if treated in early stage. Persistent high risk-human papillomavirus (HR-HPV) infection is the main cause of the development of CC. HR-HPV infection is common, especially in sexually active young women, but most infections are transient, spontaneous, and have no clinical symptom. However, 10% of women have persistent HR-HPV infection and are at risk of CC and its precursor.

HR-HPV is inclined to bring about persistent infection that has reached an agreement. To date, the studies mostly focus on the factors that contribute to persistent HPV infection. However, there is no agreement on the specific subtypes of persistent HPV infection was prone to occur. In addition, the relationship between the age and persistent HPV infection is still controversial.

In clinical practice, the study of the specific genotype of HR-HPV persistent infection is of great significance to reduce the incidence of high-grade lesions, which can be a helpful guide for the prevention and treatment of CC. Therefore, we conducted a meta-analysis to obtain the distribution and prevalence of persistent HPV infection in female worldwide, which would further optimize the prevention strategies of CC and provide reference for the development of HPV vaccines.
